# Complete Resection of a Giant Aggressive Angiomyxoma Using a Rotation Flap: A Case Report

**DOI:** 10.70352/scrj.cr.26-0367

**Published:** 2026-09-16

**Authors:** Shusaku Hiyama, Ken Imaizumi, Nobuki Ichikawa, Tadashi Yoshida, Yosuke Ohno, Kengo Shibata, Shunji Sano, Taku Maeda, Ryoji Yokoyama, Zenichi Tanei, Shinya Tanaka, Akinobu Taketomi

**Affiliations:** 1 Department of Gastroenterological Surgery I, Graduate School of Medicine, Hokkaido University, Sapporo, Hokkaido, Japan; 2 Department of Plastic and Reconstructive Surgery, Faculty of Medicine, Hokkaido University, Sapporo, Hokkaido, Japan; 3 Department of General Surgery, Abashiri-Kosei General Hospital, Abashiri, Hokkaido, Japan; 4 Department of Surgical Pathology, Hokkaido University Hospital, Sapporo, Hokkaido, Japan

**Keywords:** aggressive angiomyxoma, gluteal or perineal region, rotation flap

## Abstract

**INTRODUCTION:**

Aggressive angiomyxoma (AAM) is a rare mesenchymal tumor characterized by locally infiltrative growth and a relatively high rate of local recurrence. Surgical management requires careful planning and tailored operative strategies to achieve negative resection margins while preserving adjacent organs. We report a case of AAM treated with complete surgical resection using a rotation flap technique while preserving the surrounding organs.

**CASE PRESENTATION:**

A 58-year-old woman presented to her primary physician with elevated serum carcinoembryonic antigen levels, and physical examination revealed a subcutaneous mass on the right buttock. Needle biopsy confirmed the diagnosis of AAM. Gonadotropin-releasing hormone agonist therapy with leuprorelin was administered for 1 month; however, no tumor reduction was observed. Therefore, a surgical excision was planned. To achieve tumor removal with negative margins while preserving the surrounding organs, we collaborated with the plastic surgery team and employed a rotation flap technique, which provided excellent surgical exposure and facilitated wound closure. The lesion was completely resected with negative margins, and histopathological examination confirmed the diagnosis of AAM. The patient remains alive without evidence of recurrence 1 year postoperatively.

**CONCLUSIONS:**

The present case suggests that rotation flap reconstruction may be a useful option for the surgical management of large perineal or gluteal tumors, such as AAM. This technique allows for a flexible skin incision design, facilitates adequate surgical exposure, and enables reliable wound closure with minimal invasiveness.

## Abbreviation

AAM
: aggressive angiomyxoma

## INTRODUCTION

AAM is a rare mesenchymal tumor that typically arises from the connective tissues of the perineal and pelvic regions.^1)^ Although histologically benign, it is characterized by locally infiltrative growth and a relatively high rate of local recurrence.^1,2)^ Therefore, surgical management requires careful planning and tailored operative strategies to achieve negative resection margins while preserving adjacent organs. This report describes a case of AAM in which a surgical approach incorporating a local flap technique enabled excellent surgical exposure, thereby facilitating both complete resection and organ preservation.

## CASE PRESENTATION

A 58-year-old woman was referred to another institution after an elevated serum carcinoembryonic antigen level was detected during a routine health checkup. Physical examination revealed a subcutaneous mass on the right buttock. Needle biopsy of the lesion confirmed AAM, and the patient was subsequently referred to the Department of Gastroenterological Surgery I at Hokkaido University Hospital for surgical treatment. Contrast-enhanced CT revealed an irregularly shaped tumor measuring >10 cm in maximum diameter, extending from the subcutaneous tissue of the right buttock to the vulvar and right perianal regions, with heterogeneous enhancement (**Fig. 1A**, **1B**). MRI revealed a lesion with low signal intensity on T1-weighted images and heterogeneous high- and low-signal intensities with a characteristic swirling pattern on T2-weighted images (**Fig. 1C**, **1D**), consistent with AAM. Because the tumor exceeded 10 cm in diameter and was adjacent to the urethra, vulva, and anal canal, gonadotropin-releasing hormone agonist therapy with leuprorelin was administered for 1 month. However, no significant tumor reduction was observed, and surgical resection was therefore planned. Tumor excision with organ preservation was intended; however, conversion to abdominoperineal resection or total pelvic exenteration was considered depending on the intraoperative findings. Given the large tumor size and partial compression of the overlying skin, extensive skin defects and dead space formation were anticipated after resection. Therefore, surgery was planned in collaboration with the plastic surgeons to facilitate flap reconstruction for wound closure.

**Fig. 1:**
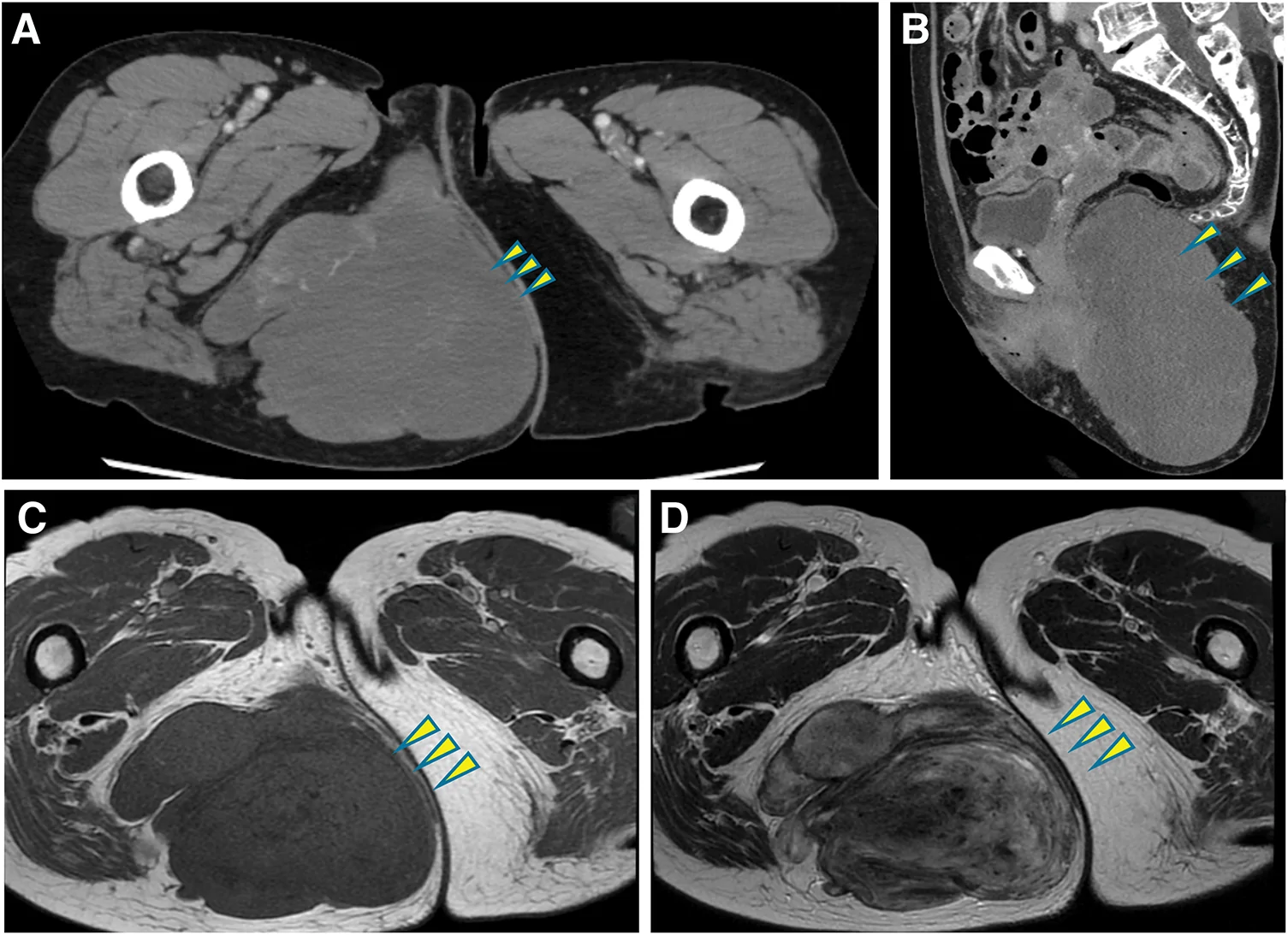
Radiological findings of the tumor. (**A**, **B**) Contrast-enhanced CT showing an irregularly shaped tumor measuring more than 10 cm in diameter in the subcutaneous tissue of the right buttock (arrowheads), extending to the vulvar and right perianal regions, with heterogeneous enhancement. (**C**, **D**) MRI demonstrating low signal intensity on T1-weighted images (**C**) and heterogeneous high and low signal intensities with a characteristic swirling pattern on T2-weighted imaging (**D**), suggestive of AAM (arrowheads). AAM, aggressive angiomyxoma

A long curvilinear incision was made from the perianal region to the lateral aspect of the right buttock (**Fig. 2A**) to provide excellent surgical exposure. Although the tumor was encapsulated, the boundaries between the tumor, the levator ani muscle, and the vagina were partially indistinct. Nevertheless, careful dissection under good visualization allowed complete macroscopic resection of the tumor without injury to the urethra, vagina, or anal canal (**Fig. 2B**–**2E**). After tumor removal, flap perfusion was quantitatively assessed using indocyanine green fluorescence angiography with a near-infrared imaging system (PDE-GEN3; Hamamatsu Photonics K.K., Hamamatsu, Japan) (**Fig. 3A**). Areas with reduced perfusion were trimmed, and the defect was reconstructed using the rotation flap technique (**Fig. 3B**). A diverting ileostomy was performed to prevent contamination of the reconstructed wound. The operative time was 6 h and 36 min, and the intraoperative blood loss was 641 mL. Postoperatively, the patient was managed on a floating bed to reduce the pressure on the reconstructed flap. The drains placed around the flap were removed on POD 5. Although partial distal flap ischemia occurred, it was successfully managed with serial debridement of the devitalized tissue and hyperbaric oxygen therapy. Once the wound bed had stabilized, negative-pressure wound therapy was applied for 2 weeks (POD 26–42) to promote healthy granulation tissue formation and progressive wound healing. The patient was discharged on POD 49 after achieving satisfactory wound healing. The resected specimen weighed 1011 g and measured 160 × 150 × 90 mm (**Fig. 4**). It was a grayish-white, lobulated mass with irregular margins. Histopathological examination revealed the proliferation of spindle-shaped cells within a fibromyxoid stroma, accompanied by numerous dilated blood vessels and focally hypercellular nodules (**Fig. 5A**). Immunohistochemical staining demonstrated positivity for α-smooth muscle actin, desmin, estrogen receptor, CD34, and CDK4 (**Fig. 5B**–**5F**), consistent with the preoperative biopsy findings and confirming the diagnosis of AAM. No histological evidence of a therapeutic response to preoperative leuprorelin was observed. All the resection margins were negative. Ileostomy closure was performed 7 months after the initial surgery, and the patient remained recurrence-free at 1-year follow-up (**Fig. 6**).

**Fig. 2:**
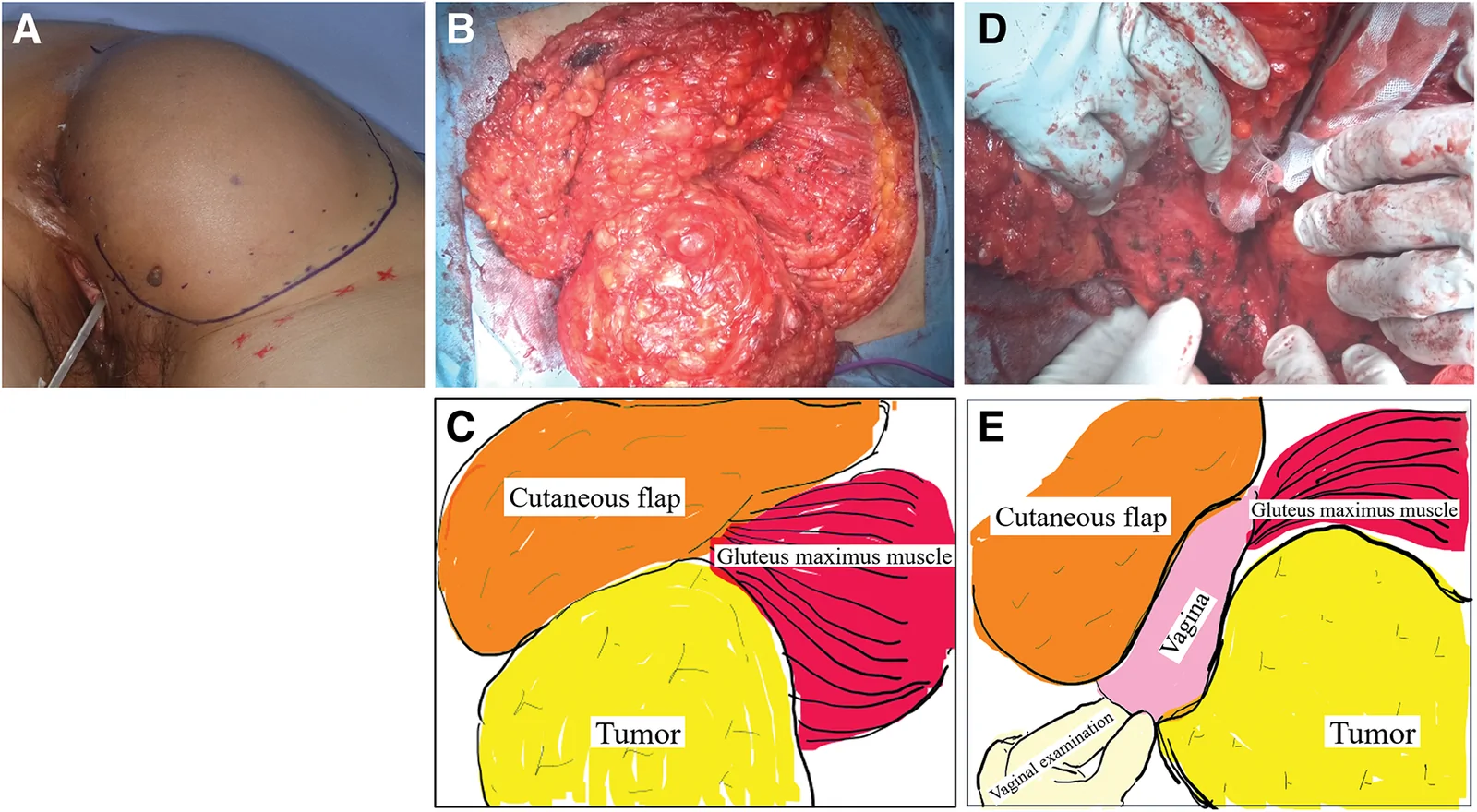
Intraoperative findings and schematic illustrations of the surgical procedure. (**A**) An arcuate skin incision was made in the right buttock. A relatively large incision was designed in collaboration with plastic surgeons to obtain an adequate operative field. (**B**, **C**) The tumor was carefully dissected from the gluteus maximus muscle. The corresponding step is illustrated schematically in panel (**C**). (**D**, **E**) On the medial side, digital rectal and vaginal examinations were performed simultaneously to identify the boundary between the tumor and the anal canal and vagina, enabling safe dissection. The corresponding step is illustrated schematically in panel (**E**).

**Fig. 3:**
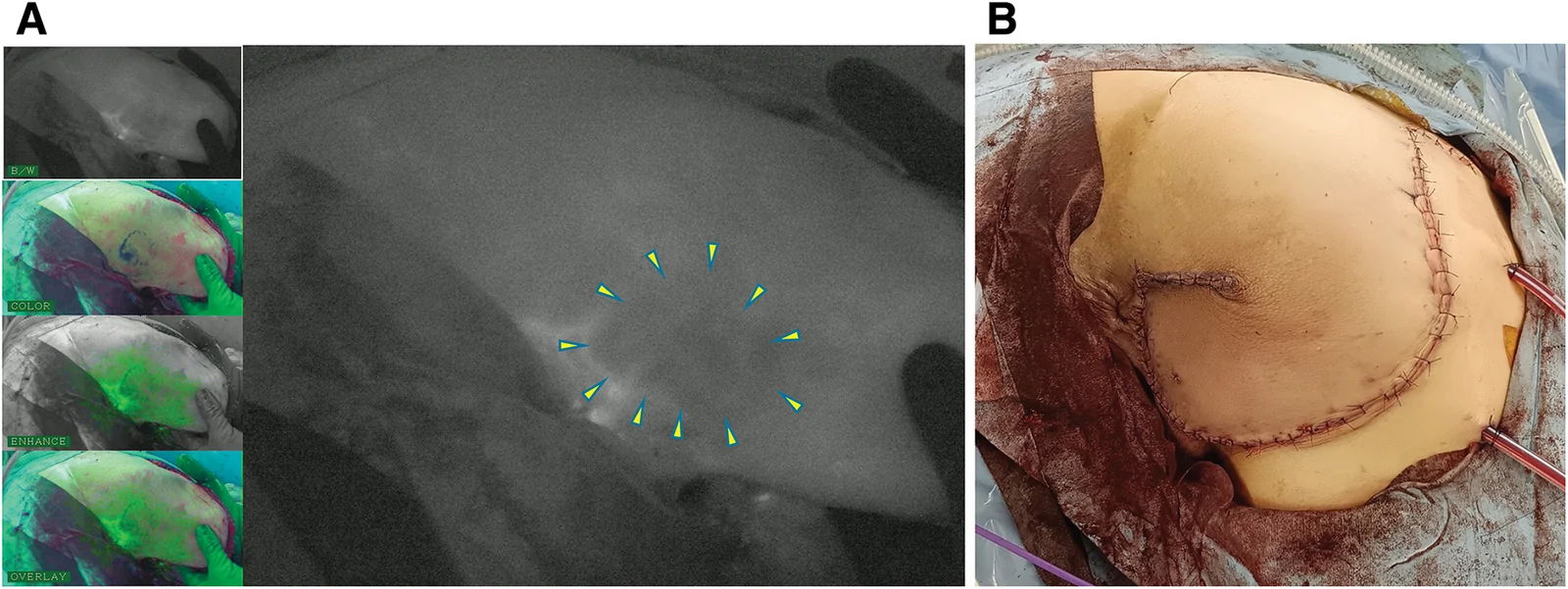
Intraoperative assessment of flap perfusion and wound closure. (**A**) ICG fluorescence imaging using a near-infrared imaging system (PDE-GEN3; Hamamatsu Photonics K.K., Hamamatsu, Japan) was used to assess flap perfusion after intravenous injection of 5 mg of ICG. Areas with decreased perfusion (arrowheads) were trimmed to ensure adequate vascularity. (**B**) The wound was subsequently closed using a rotation flap technique. ICG, indocyanine green

**Fig. 4:**
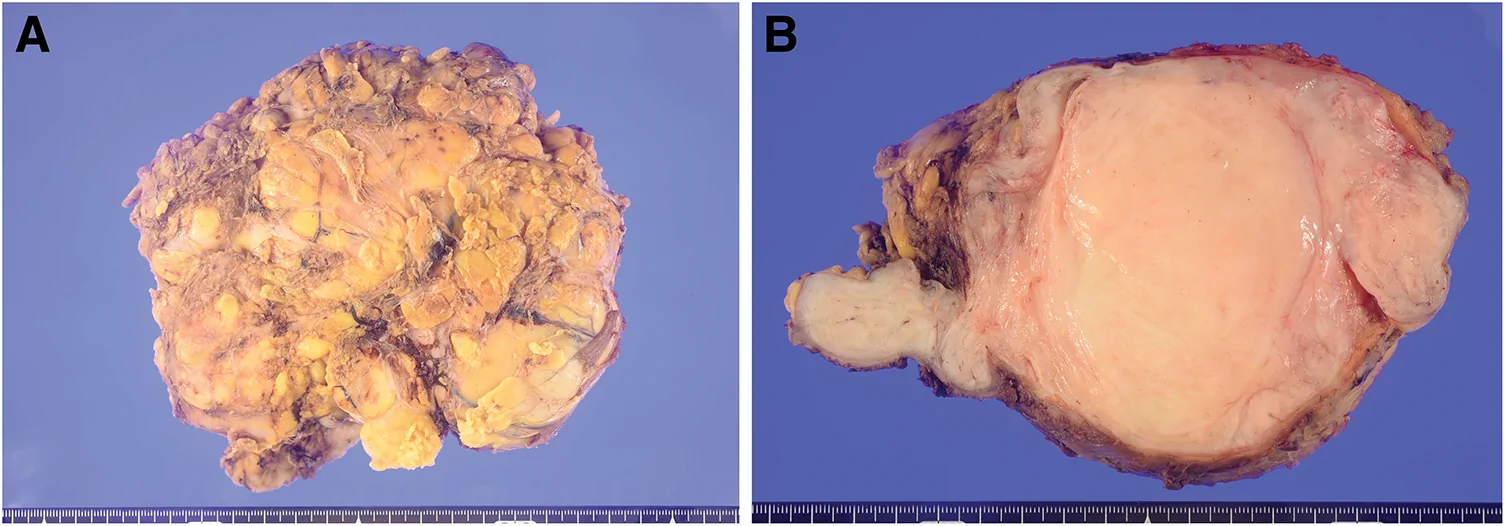
Gross appearance of the resected specimen. (**A**) The resected tumor measured 160 × 150 × 90 mm and weighed 1011 g. (**B**) The specimen showed a lobulated grayish-white mass with irregular margins.

**Fig. 5:**
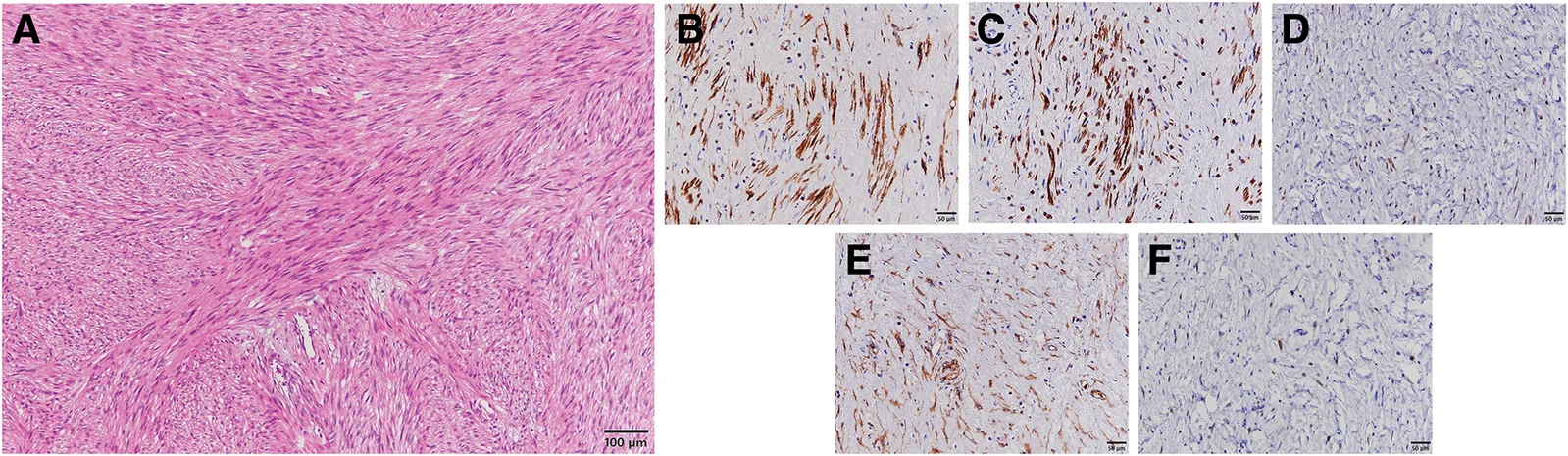
Histopathological findings. (**A**) Hematoxylin and eosin staining showed proliferation of spindle-shaped cells within a fibromyxoid stroma containing numerous dilated blood vessels. Focally increased cellularity forming nodular areas was observed (×10). (**B**–**F**) Immunohistochemical staining (×20) demonstrated positivity for (**B**) α-smooth muscle actin, (**C**) desmin, (**D**) estrogen receptor, (**E**) CD34, and (**F**) CDK4, findings consistent with AAM. AAM, aggressive angiomyxoma

**Fig. 6:**
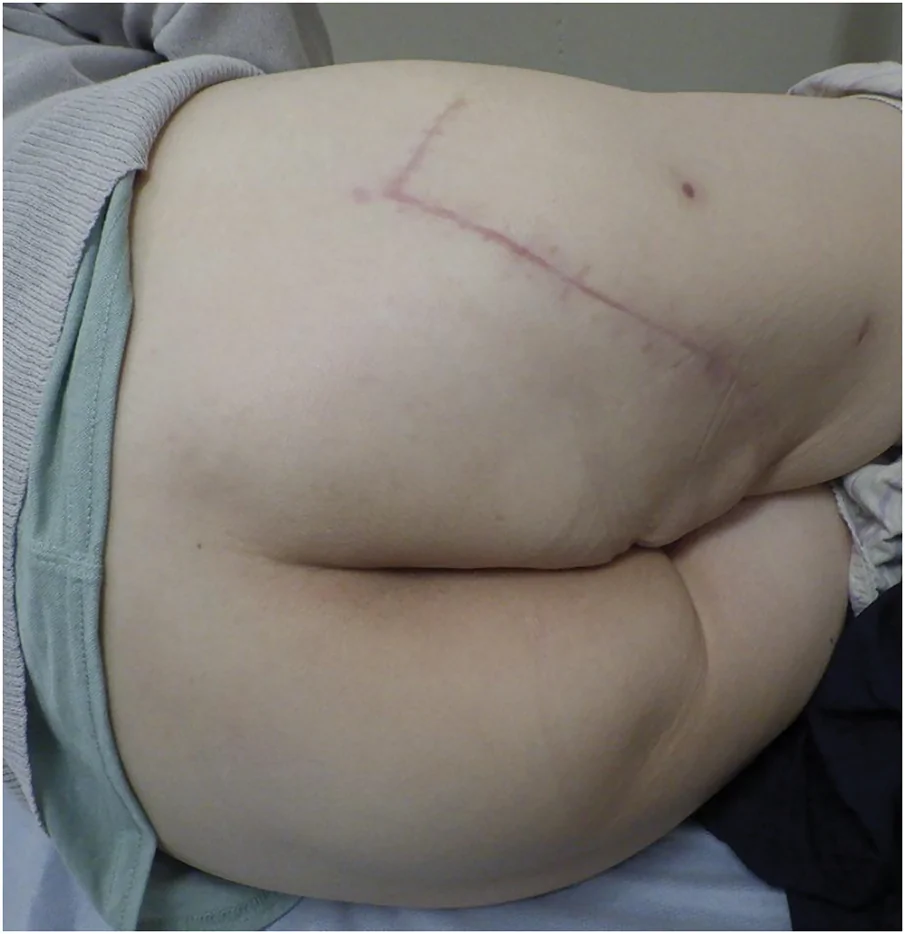
Postoperative appearance of the wound at outpatient follow-up. Photograph of the surgical wound 6 months after surgery showing favorable wound healing without ischemia or scar contracture.

## DISCUSSION

In the present case, a 58-year-old woman with a giant AAM measuring >10 cm in the gluteal region underwent surgical resection in collaboration with the plastic surgery team. An excellent operative field was obtained by planning reconstruction with a rotation flap in advance and creating a wide skin incision. This approach allowed careful evaluation of the relationship between the tumor and surrounding organs and enabled safe dissection under direct visualization, achieving both organ preservation and negative surgical margins. The postoperative course was generally acceptable. Partial flap ischemia developed but improved with conservative wound care without progression to flap necrosis or surgical site infection. Although various reconstructive techniques have been reported for large perineal defects, reports describing the planned use of local rotation flaps, specifically for giant AAM, remain limited. Rather than representing novelty of the disease, this case highlights the practical usefulness of incorporating reconstructive planning into the initial surgical strategies. This approach achieved both adequate exposure and organ preservation while enabling reliable wound closure and may represent a useful reconstructive option for selected giant tumors involving the buttock and perineal regions.

AAM was first described by Steeper and Rosai in 1983 as a myxoid tumor characterized by prominent angiogenesis, locally infiltrative growth, and a high propensity for local recurrence.^1)^ Although AAM is relatively rare, >500 cases have been reported worldwide. It predominantly affects women in the second to fourth decades of life, with a reported female-to-male ratio of approximately 6:1.^3)^ The most common sites of occurrence are the pelvic cavity, perineum, and the perianal region. Among these, MRI is considered the most useful. Typical findings include low signal intensity on T1-weighted images and high signal intensity on T2-weighted images, with strong enhancement after gadolinium administration, demonstrating a characteristic swirling or layered internal architecture.^4)^ Histopathologically, AAM is characterized by a hypocellular myxoid stroma containing numerous blood vessels of varying calibers, along with small spindle-shaped or stellate tumor cells showing minimal nuclear atypia and inconspicuous mitotic activity.^5)^ On immunohistochemistry, tumor cells variably express vimentin, desmin, CD34, smooth muscle actin, estrogen receptor, and progesterone receptor; however, none of these markers is specific to AAM.^6)^ Surgical excision remains the first line of treatment for AAM. Luteinizing hormone-releasing hormone agonists have been reported to be effective in patients with hormone receptor positivity.^7)^ Distant metastasis is extremely rare,^8,9)^ and the overall prognosis is favorable.^8)^ Nevertheless, local recurrence is relatively common, with most recurrences occurring within 5 years after surgery,^3)^ although the timing of recurrence varies widely. Kiran et al. reported a case of recurrence as late as 20 years after initial surgery, highlighting the importance of long-term postoperative surveillance.^10)^

Hormonal therapy with gonadotropin-releasing hormone agonists has been reported to reduce the tumor size in some patients with estrogen receptor–positive AAM and may facilitate subsequent surgical resection. However, the response to hormonal therapy is variable, and the optimal duration of preoperative treatment has not been established.^7,11,12)^ In the present case, no measurable reduction in tumor size was observed after 1 month of leuprorelin administration. Because complete surgical resection was considered technically feasible despite the large tumor size, and the patient preferred definitive surgical treatment, prolonged neoadjuvant hormonal therapy was not pursued.

Although surgical resection is generally regarded as the first line of treatment for AAM, no standardized surgical strategy has been established. Previous reports have described various approaches, including combined laparoscopic and perineal resections for AAM,^13)^ as well as cases in which pelvic exenteration was selected for giant tumors with invasion into adjacent organs.^14–17)^ Furthermore, because tumor margins are often ill-defined on both palpation and visual inspection, achieving clear surgical margins can be technically challenging, with a reported rate of microscopically positive margins of approximately 36%.^18)^ Therefore, the surgical management of AAM requires careful consideration of the operative approach and the extent of resection to achieve negative margins while preserving the surrounding organs. In the present case, preoperative CT demonstrated that the tumor was in close proximity to the adjacent organs, including the urethra, vagina, and anal canal, and exceeded 10 cm in diameter. Consequently, in the present case, there was a potential risk of combined resection of adjacent organs, as well as extensive skin defects and dead space formation following tumor excision. To address these concerns, a rotation flap using well-vascularized surrounding tissue was employed, enabling the effective obliteration of dead space and secure wound closure. In addition, preoperative planning with reconstruction allowed for adequate skin incision design, providing excellent surgical exposure. The schema illustrates the process from skin incision to flap blood flow evaluation, trimming, and wound closure (**Fig. 7**). This approach likely contributed to safe tumor resection, organ preservation, and the achievement of negative surgical margins.

**Fig. 7:**
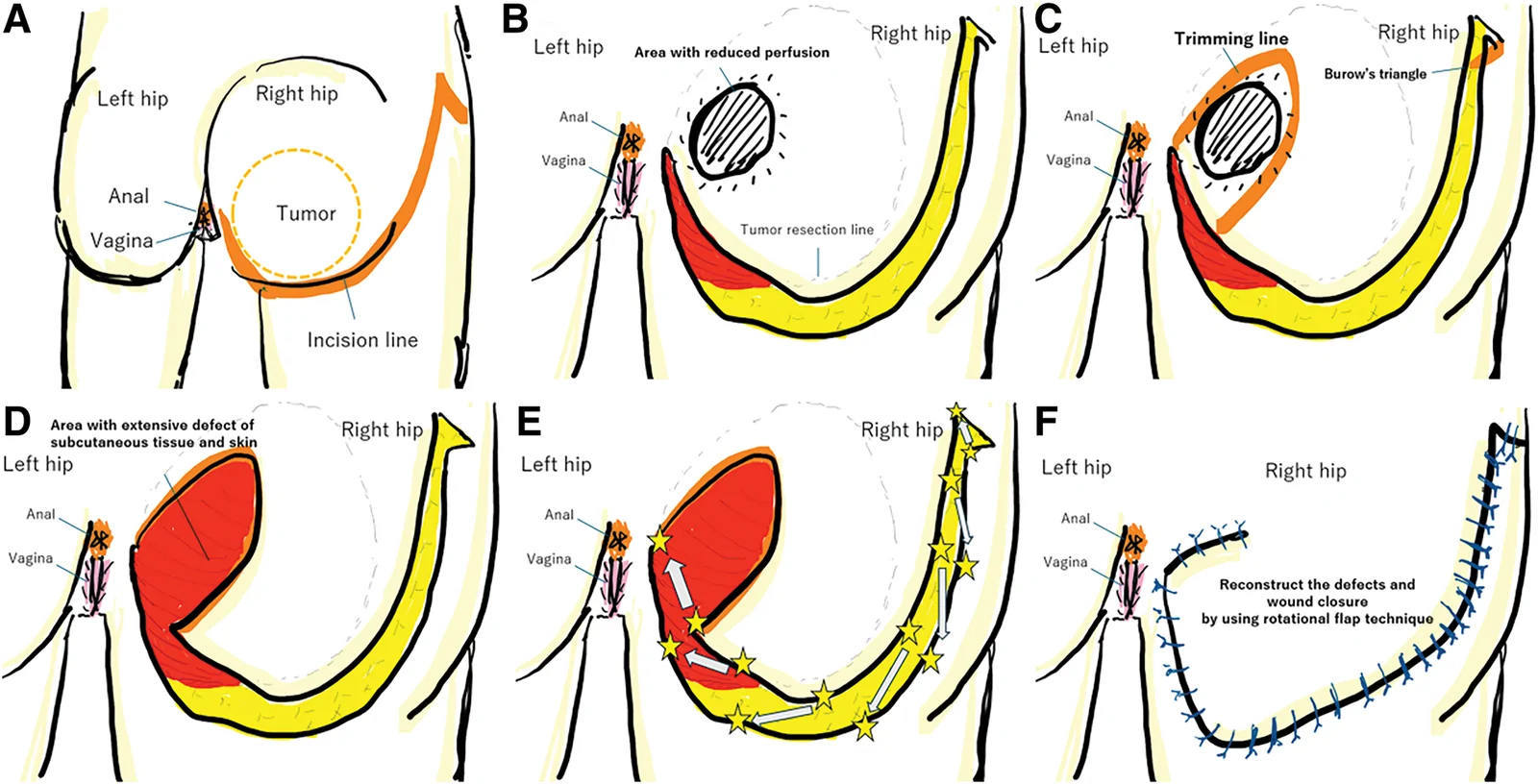
Schematic illustrations of the surgical procedure from skin incision to flap blood flow evaluation, trimming, and wound closure. (**A**) The design of the incision line. (**B**) Flap perfusion was assessed by indocyanine green fluorescence angiography. (**C**) The flap area with reduced perfusion was trimmed. A Burow’s triangle was excised to facilitate tension-free closure and avoid dog-ear formation. (**D**) After trimming, an extensive defect of the subcutaneous tissue and skin was observed. (**E**) Images illustrating reconstruction using a rotation flap. (**F**) The defect was reconstructed and the wound was closed using a rotation flap technique.

The reconstructive strategy was based on the concept of a reconstructive ladder, which selects the least invasive technique capable of achieving reliable wound closure while preserving more complex reconstructive options, if required in the future.^19)^ Given the anticipated size of the skin defect, achieving a satisfactory coverage of the primary closure or skin grafting was considered unlikely; therefore, flap reconstruction was planned. Various reconstruction options are available for perineal and gluteal defects, including local, musculocutaneous, and perforator flaps.^20,21)^ Musculocutaneous flaps provide sufficient tissue to obliterate extensive dead space,^22)^ whereas perforator flaps preserve muscle function but are technically more demanding.^20,23)^ In the present case, the tumor was confined to the subcutaneous tissue without muscle invasion, and the residual dead space was not considered large enough to require a musculocutaneous flap. Furthermore, because AAM is associated with a substantial risk of local recurrence, preserving future reconstructive options was also considered during surgical planning, as extensive flap reconstruction during the initial operation may limit reconstructive choices when repeat surgery becomes necessary. Among the local flap options, both the V–Y advancement flap and rotation flap were considered. However, the final extent of skin excision depends on the intraoperative assessment of the tumor margins and skin viability, necessitating a flexible flap design. Furthermore, a wide surgical exposure is essential to facilitate meticulous dissection along the appropriate tissue plane while preserving the urethra, vagina, and anal canal. Compared with the V–Y advancement flap, the rotation flap provided greater flexibility in flap design and allowed wider operative exposure while enabling tension-free closure of the anticipated skin defect. These considerations supported the selection of a rotation flap as the most appropriate reconstruction strategy in the present case.

In addition to careful reconstructive planning, temporary fecal diversion is considered an important adjunctive strategy for protecting reconstructed perineal wounds during the early postoperative period. Although no macroscopic rectal injury was identified intraoperatively, extensive dissection adjacent to the rectal wall was required because the tumor was large and its boundary with the surrounding normal tissue was indistinct. Consequently, fecal diversion was also considered appropriate to minimize the potential consequences of an occult rectal injury should it become clinically apparent postoperatively.^24)^ Furthermore, although the anal sphincter complex was preserved, extensive resection of the surrounding perianal soft tissue raised concerns that transient postoperative anorectal dysfunction could result in fecal soiling of the reconstructed wound. Temporary fecal diversion has also been reported to facilitate management after extensive perineal reconstruction.^25)^ Therefore, a diverting ileostomy was performed to reduce the risk of fecal contamination, protect the rotation flap, and facilitate postoperative wound management. In routine clinical practice, stoma reversal is often delayed by several months, although earlier stoma closure has recently been investigated in selected patients, and the optimal timing remains controversial.^26)^ Because the fecal diversion in the present case was intended to protect the reconstructed perineal wound rather than the colorectal anastomosis, stoma closure was individualized according to the postoperative course. The ileostomy was successfully closed 7 months after surgery, following confirmation of complete wound healing and satisfactory anorectal function.

AAM is a relatively rare disease, and many aspects of its biological behavior and pathogenesis remain unclear. While some reports suggest that achieving negative surgical margins contributes to improved local control, others have shown no significant difference in recurrence-free survival within 10 years after surgery between patients with positive and negative surgical margins.^3)^ Therefore, further studies are required to better elucidate the biological characteristics of this tumor and clarify the true impact of margin status on the risk of recurrence.

A surgical strategy incorporating plastic reconstruction techniques, as employed in the present case, may represent a useful option for achieving safe and potentially curative resection under optimal operative exposure in patients with AAM and other giant tumors of the perineal or gluteal region.

## CONCLUSIONS

The present case suggests that rotation flaps may represent a useful reconstructive option for the surgical management of perineal and gluteal tumors such as AAM. This technique enables a flexible skin incision design that facilitates surgical exposure while allowing reliable wound closure with minimal additional invasiveness. Although complete tumor resection with preservation of the adjacent organs was successfully achieved in the present case, long-term follow-up is warranted because AAM is associated with a risk of late local recurrence.

## DECLARATIONS

### Availability of data and materials

The data supporting the findings of this study are available from the corresponding author upon request.
